# Sequence locally, think globally: The Darwin Tree of Life Project

**DOI:** 10.1073/pnas.2115642118

**Published:** 2022-01-18

**Authors:** The Darwin Tree of Life Project Consortium

**Keywords:** genome, sequencing, biodiversity, assembly

## Abstract

The goals of the Earth Biogenome Project—to sequence the genomes of all eukaryotic life on earth—are as daunting as they are ambitious. The Darwin Tree of Life Project was founded to demonstrate the credibility of these goals and to deliver at-scale genome sequences of unprecedented quality for a biogeographic region: the archipelago of islands that constitute Britain and Ireland. The Darwin Tree of Life Project is a collaboration between biodiversity organizations (museums, botanical gardens, and biodiversity institutes) and genomics institutes. Together, we have built a workflow that collects specimens from the field, robustly identifies them, performs sequencing, generates high-quality, curated assemblies, and releases these openly for the global community to use to build future science and conservation efforts.

## Sequence Everything

The 300-y Linnaean project, the explicit naming in a global *lingua franca* of all species on Earth, is one of humanity’s great successes. Over 2 million (of an expected >10 million) species have been formally named (1). This Linnaean namespace underpins all activity in comprehension of the natural world, in conservation, and in biocommerce. The need for understanding is critical, as climate change, globalization of trade, and the degradation of agricultural and natural habitats drive the sixth mass extinction, and with it the productivity on which humans depend (2). The looming need to establish postoil economies and the promise of new feedstocks for bioindustry demand deeper exploration of the biosphere. We need novel medicines to combat emerging and resurgent diseases, and the natural pharmacopoeia has much to offer in the form of novel compounds. Openly accessible understanding of species’ biology is a global good.

All life on Earth is connected by the common thread of DNA, modified through the engines of mutation, selection, and drift to generate Darwin’s “endless forms” (3). The processes of evolution, speciation, and adaptation are continuing in the world around us and the patterns left in DNA can be used to reconstruct life’s history and understand the mechanisms behind the origins of diversity. The functional sequences in living species, honed by 3.5 billion y of evolution, represent a vast natural experiment in finding the diverse ways in which protein and RNA molecules can catalyze biotransformations to generate a universe of active molecules.

The Earth BioGenome Project (EBP) proposes that all known eukaryotic species on our planet should be sequenced as a resource for future bioscience and environmental stewardship (4). This decadal project will deliver the raw material for a new approach to our biological understanding of the natural world. If the Linnaean system is a universal catalog of the books of life, the EBP will fill the pages of every volume with base-level precision and evolutionary stories not yet imagined. The goals of the EBP will be delivered through a global collaboration of groups generating data from species selected by taxonomy or location. We have initiated a project to sequence to high quality the genomes of all known eukaryotic species in a defined geographical region: the islands of Britain and Ireland. Here we describe this Darwin Tree of Life Project (DToL).

## Why Sequence the Genomes of Species in Britain and Ireland?

Britain and Ireland have a centuries-old tradition of natural history recording and of landscape-scale ecological science, embodied in long-term ecological research infrastructures and national recording organizations, globally significant museums and botanic gardens, campaigning societies that aim to protect the natural world, and expert natural history societies with a regional or taxonomic focus.

Our Atlantic archipelago has a maritime climate formed by the conflicting drivers of the North Atlantic Drift and Gulf Stream on the west and south, and the Norwegian Current and the basin of the North Sea to the east and north. These influences impact on a wide range of marine, terrestrial, and freshwater habitats. The terrestrial biota of these islands is largely the result of recolonization since the last glacial maximum (5, 6), conditioned by underlying geological and climatic influences. From western temperate rain forests to southern chalk downlands and fens and marshland on the eastern seaboard, British and Irish ecology is a model for the effects of and responses to climate change. The terrestrial biota is relatively depauperate compared to the European continent, with very few endemic species (7). However, many species are at the northern edge of their ranges and have strongly fragmented populations. In the marine realm the biota is dominated by the currents that control terrestrial climate and also by the contrast between the highly fragmented, glacially sculpted west coast swept by the northern reach of the Gulf Stream and an east coast typified by postglacial isostatic rebound and deposition. The archipelago lies on a biogeographic transition zone between Boreal and Lusitanian marine provinces, and the leading or trailing distributional range limits of many marine species from both provinces occur in this region. It is already evident that species on these islands are responding to climate change, with new records established for northern ranges and new species naturally colonizing from the continent, making them a useful laboratory in which to observe the effects of rapid climate change. Britain and Ireland are also subject to invasion, a process that is likely to be speeding up, and which has unpredictable effects on native taxa (8, 9). The biota has also been subject to thousands of years of anthropogenic impact in terms of deforestation, agricultural development, and pollution. However, and importantly, despite low overall species diversity, the biota has representatives from a wide range of taxonomic groups: 60% of all orders of Eukaryota, 40% of families, and 25% of genera (Table 1).

**Table 1.** The eukaryotic biota of Britain and Ireland

Kingdom	Phyla	Classes	Orders	Families	Genera	Species
All UKSI taxa	63	218	880	**4,174**	19,257	72,572
Fungi	7	35	166	**578**	3,198	18,512
Metazoa	32	96	405	**2,827**	13,169	41,613
Viridiplantae	9	38	156	**438**	2,083	8,072
Chromista and other microbial eukaryotes	15	49	153	**331**	807	4,375
Global taxa	70	274	1,295	**10,735**	175,901	1,879,687
DToL proportion	0.90	0.80	0.68	0.39	0.11	0.04

Data from the Natural History Museum UK Species Inventory (UKSI; https://www.nhm.ac.uk/our-science/data/uk-species.html) and Species2000 Catalogue of Life (https://www.catalogueoflife.org/) (for Global taxa) (1). Despite their apparent precision, these numbers should be regarded as first estimates. DToL initially aims to sequence one representative of each taxonomic family (bold numbers in column "Families").

Given this wealth of knowledge and expertise, we (see list of members in Table 2) have built a project that has the uniquely well-known biota of Britain and Ireland at its heart. Just as Darwin came to understand all of life’s evolution through work he carried out at his house in Downe, Kent, we believe that study of the biota of these islands will be a powerful step in understanding global genomic diversity.

**Table 2.** The partners in the Darwin Tree of Life Project

Partner	Roles in project	Location	Lead Investigators
Natural History Museum	Sample collection (especially terrestrial animals); Archiving; DNA barcoding; Analysis	London, UK	Ian Barnes Gavin Broad
Royal Botanic Garden Edinburgh	Sample collection (plants and lichens); Archiving; DNA barcoding; Analysis	Edinburgh, UK	Michelle Hart Peter Hollingsworth
Royal Botanic Gardens Kew	Sample collection (plants and fungi); Archiving; DNA barcoding; Analysis	London, UK	Paul Kersey Ester Gaya
Marine Biological Association	Sample collection (marine and littoral species); DNA barcoding; Analysis	Plymouth, UK	Nova Mieszkowska Willie Wilson
University of Oxford	Sample collection (Wytham Woods genomic observatory; protists); Analysis	Oxford, UK	Peter Holland Thomas Richards Owen Lewis
Earlham Institute	Sample collection (protists); Data infrastructure; Sequencing (protists); Analysis	Norwich, UK	Neil Hall Rob Davey
Wellcome Sanger Institute	Sample collection; Data infrastructure; Sequencing; Assembly; Analysis	Cambridge, UK	Mark Blaxter Matt Berriman Mara Lawniczak
University of Edinburgh	Sequencing; Assembly; Analysis	Edinburgh, UK	Alex Twyford
University of Cambridge	Assembly; Analysis	Cambridge, UK	Richard Durbin
EMBL-EBI	Databasing; Data infrastructure; Analysis	Cambridge, UK	Paul Flicek Kevin Howe
Connecting Science	Outreach and engagement	Cambridge, UK	Kenneth Skeldon

## Why Sequence All Life and Not Just Representative Species?

We propose, ultimately, to sequence all species in and around these islands, not just a “representative” few. This goal is not driven by a completist mentality but rather a realization that many of the features of genomes that are of interest can only be discovered through close sister species comparison and that the nature of species and the correlates of species boundaries can only be measured if comprehensive data are available. Similarly, patterns and processes that generate and maintain genomic diversity can only be described using data from across the phylogenetic diversity of life. The sets of questions that can be fruitfully addressed using complete genomes across life are legion (10).

Our first goal is to deliver a reference genome assembly for every taxonomic family represented in Britain and Ireland (about 4,200 genome sequences). This will contribute to DToL goals and also to the goals of other major international projects, such as the Vertebrate Genomes Project (adding family and ordinal reference genomes) (11), the Insects 5k initiative (30% of the species in Britain and Ireland are arthropods) (12), and the 10k Plants Genomes Project (13). From there we will proceed to generate references for all genera and then all described species. While we aim to complete the biota, it is evident that doing some species early will yield disproportionate returns. We will further prioritize species if they are iconic (e.g., are used to represent an ecosystem or group, and have strong public recognition), interesting (e.g., they are currently the subjects of investigation because of striking phenotypes or landscape ecology), or important (e.g., they are keystone species in ecosystems, or are expected to be particularly revealing of particular processes).

As noted above, much of the planet’s biodiversity has yet to be formally described, and we fully expect that there will be undescribed species in Britain and Ireland. For example, there are only ∼4,300 species of microbial eukaryote on the list of British and Irish species, but we expect that there will be a wealth of novel species and currently unrepresented higher taxa in soils and waters (14). We will explore the diversity in eukaryotic microbes in freshwater and marine habitats using single-cell or microculture genomics (15) and bulk long-read and long-range metagenomics methods. We are also developing methods to sequence very small organisms, such as sediment meiofauna and the numerous miniscule insects, from single specimens (16).

## Why Sequence Whole Genomes?

We intend to sequence whole genomes and to generate chromosomally complete assemblies wherever possible. This goal derives from our confidence, given the advances in data generation and assembly technologies, that we will be able to achieve this goal for the vast majority of the species we analyze. Collecting and identifying specimens for sequencing is a major task in itself, and recollecting at the scale we propose is not likely to be easily repeatable; we need to do the sequencing from these valuable specimens once and do it well. We aim for chromosomally complete assemblies because we are interested in the biology of the whole genome, not just the coding genes. Genomes have long-range structures that have major effects on gene expression and which evolve through phylogenetic time. While we cannot yet fully interpret any of the genomic books of life, our ability to decipher each one will depend on its completeness and correctness.

We contend that the time to sequence all genomes is now because of a confluence of opportunities: a step change in sequencing methods and a step change in analysis algorithms. Long-read technologies—from Pacific Biosciences and Oxford Nanopore—are capable of generating single reads hundreds of thousands of bases long. The Pacific Biosciences high-fidelity (HiFi) or circular consensus mode produces 15- to 20-kb reads of similar accuracy to standard Illumina short reads. Importantly, new instruments from both Pacific Biosciences and Oxford Nanopore mean that the per assembled gigabase cost is now very reasonable. To complement long reads, new ways of scaffolding sequences into chromosomes using long-range data have been developed. Chromosomes within a nucleus are not randomly dispersed, but are folded into metastable domain structures. Methods that use proximity ligation of in situ cleaved chromosomal DNA (Hi-C and related methods) generate data that can be used to stitch sequence contigs into chromosomes.

In parallel, there has been radical innovation in assembly algorithms that deliver better results using less computer power (both computation and memory) and less time (17, 18). Long, high-quality reads span and resolve most repeat elements, and the contiguity of primary assemblies from individual specimens is tens to thousands of times that of short-read equivalents. Hi-C data robustly generate scaffolds that correspond to chromosomes or chromosome arms (19), and judicious, evidence-driven curation corrects remaining errors and links scaffolds to deliver final assemblies where the vast majority of the sequence is in chromosomal pseudomolecules (20).

## Biodiversity Genomics at Scale: From Sample to Genome

**Fig. 1.**
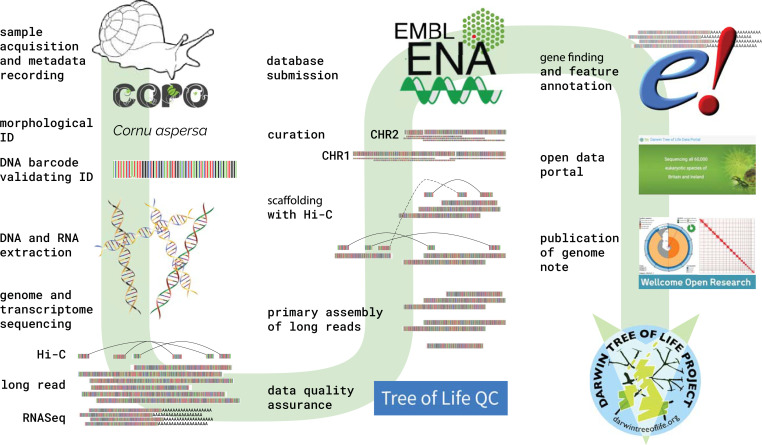
Sequencing eukaryotic life at scale. The DToL partners have developed a cohesive end-to-end process to take organisms from the field through to publication of high-quality assembled and annotated genomes in the public domain. By collating specimen metadata and tracking information through identification, DNA barcoding, extraction, sequencing, assembly, curation, annotation, and submission, the process assures that genomes are published with rich, informative, and accurate descriptors.

Moving from genome sequencing of a few species to the sequencing of all species requires a close collaboration between diverse communities of researchers. DToL brings together the unique and complementary expertise of organizations working in biodiversity, sequencing, genomics, and analysis (Table 2), and we have crafted an end-to-end process that assures integrity, accuracy, and quality in our goals (Fig. 1).

The people who know what species are, where they are, and how to collect them, are biodiversity scientists. In DToL we build on the hundreds of years’ experience of the Natural History Museum London, the Botanic Gardens Kew, the Royal Botanic Garden Edinburgh, and the Marine Biological Association, and these organizations’ networks of professional and amateur collectors, to access species legally and ethically. We also collect from sites that we hope will become genomic ecological observatories, especially the University of Oxford’s long-term ecological field site, Wytham Woods (21). Developing a standard set of specimen metadata has been a central task for the project. We have defined a Sampling Code of Practice (https://zenodo.org/record/5602825) that emphasizes our requirement to have documented provenance for every specimen and to have minimal environmental impact, and developed standard procedures for metadata collection and secure cold-chain shipping.

Live specimens are identified, photographed, and carefully recorded. Because species can be hard to identify, we also DNA barcode all specimens at mitochondrial, chloroplast, or ribosomal RNA loci before proceeding to genomic analysis. While the DNA barcode library for British and Irish species is incomplete, this step serves to affirm identifications made in the field, and to generate a sequence tag to track specimens as they are processed further. It also highlights issues where collectors’ species concepts differ and will identify potentially cryptic taxa.

To coordinate all this work, from field collection to sequence deposition, we have developed sample- and data-tracking systems. The rich metadata we collect for each specimen is collated and shared in a project database hosted by the Earlham Institute’s COPO infrastructure (https://copo-project.org/). These data then feed forward to the sequencing and analysis teams, and to a public portal (https://portal.darwintreeoflife.org/). To better estimate the amount of data needed per species, and the expected chromosomal number, we are remeasuring C-values and chromosome numbers in many taxa (especially plants) (22) and have collated available karyotype and genome size data across eukaryotes to better estimate these values for new species in an openly accessible, searchable portal, Genomes on a Tree (https://goat.genomehubs.org).

Specimens are flash-frozen and shipped to the Wellcome Sanger Institute for processing and sequencing. We are developing rapid and effective procedures for extracting very long DNA (modal fragment lengths above 150 kb) and high-quality RNA from individual specimens of all species. Particular challenges are posed by the biochemistry of some groups (such as plants and their cell walls, or the mucus present in many marine invertebrates) and by specimens that are very small (where the total number of cells, and thus the total mass of DNA available from one specimen, is below current sequencing library input requirements) (16). From the long DNA we construct long-read and long-range sequencing libraries. We aim to generate 25- to 30-fold coverage in long-read and 50- to 100-fold coverage in long-range data for each species. We also generate transcriptomic data for the majority of species, for gene finding during annotation.

## Genome Assembly at Scale

The sequencing data are subject to extensive quality assessment (which is released publicly: see https://tolqc.cog.sanger.ac.uk). Many organisms will have expected or unexpected fellow travelers, such as parasites, intracellular symbionts, and other microbiota (20, 23). If detected, we separate the data derived from these cobionts for independent assembly. Data for organellar genomes (mitochondrial and plastid) are also separated and assembled. The nuclear genome of the target species is assembled using best-practice approaches and scaffolded with the long-range data. These primary assemblies are then curated to improve the quality of the scaffolding to achieve chromosomal completeness and remove any remaining errors (20). Currently, we curate only a primary haplotype for each sequence, but also identify the sequences corresponding to secondary haplotype, necessarily less-well assembled. Both primary and secondary haplotypes, and all raw data, are submitted to the European Nucleotide Archive (ENA) (24). Once in the ENA, the genomes are processed by the European Molecular Biology Laboratory (EMBL) European Bioinformatics Institute’s Ensembl team. Ensembl is a flagship database for genomes (25) and, like other parts of the DToL process, is transforming itself to work at the scale demanded by high-throughput biodiversity genomics. Using transcriptome data, Ensembl systems find repeats, predict gene models, and annotate the features found with rich functional and comparative information (http://projects.ensembl.org/darwin-tree-of-life/). The DToL data products are presented in the public portal, linking specimens, species, and genomes (https://portal.darwintreeoflife.org).

DToL includes research at every stage in the process, including especially in informatics and data processing. DToL collaborators are working on improving assembly, and especially in exploiting the full value inherent in the new long-read and long-range data, including for example, methylation signal present in long-read data and topologically associated domain signal present in Hi-C data. The protist team is exploring use of bulk and single-cell technologies in assembling the often large genomes of the diversity of eukaryotic microbes. We are developing modular, automated workflows that permit high-throughput while also recording and reporting key quality data. As tools and processes evolve, so will our workflows.

## Open Data, Current Progress, and Future Prospects

DToL will produce openly accessible genome sequences at a huge scale. We will share our protocols for collection, extraction, and sequencing with the global community. Tree of Life quality-control data are reported openly (https://tolqc.cog.sanger.ac.uk), and project assemblies, raw data, and metadata are aggregated in a dedicated DToL portal (https://portal.darwintreeoflife.org) and annotated genomes aggregated in a dedicated Ensembl portal (http://projects.ensembl.org/darwin-tree-of-life/). Our sampling, processing, and assembly processes and software toolkits are openly available and immediately reusable in other large-scale biodiversity projects.

We are releasing assemblies as we generate them. These assemblies carry no embargo. To promote their use, and to ensure that credit accrues directly to those who have been involved in individual species’ projects, each genome will be accompanied by a genome note: a short, definitive publication announcing the availability of the genome and reporting the key specimen metadata and assembly metrics (collated at https://wellcomeopenresearch.org/treeoflife) (26–36). We will collaborate widely to analyze these genomes in evolutionary, ecological, and conservation contexts, to ensure they achieve the impact we believe they should. We hope and expect that these genomes will then be used by communities of evolutionary, ecological, conservation, and biotechnology scientists to carry out large-scale investigations of species and species groups, and we prioritize species for sequencing based on requests from these groups. From the genomes released already, we are aware of reuse by projects investigating population dynamics in threatened predator species (37), in fish stock monitoring, in lepidopteran speciation dynamics, in ancient environmental DNA analyses, and in large-scale phylogenetics. Additionally, we are developing a rich program of outreach and engagement work bringing DToL to and getting feedback from a wide diversity of stakeholders. Beyond the genomes, all remaining biological material from specimens and samples (including a set-aside aliquot of high molecular weight DNA) collected for DToL will be stored for future research in the national collections.

**Fig. 2.**
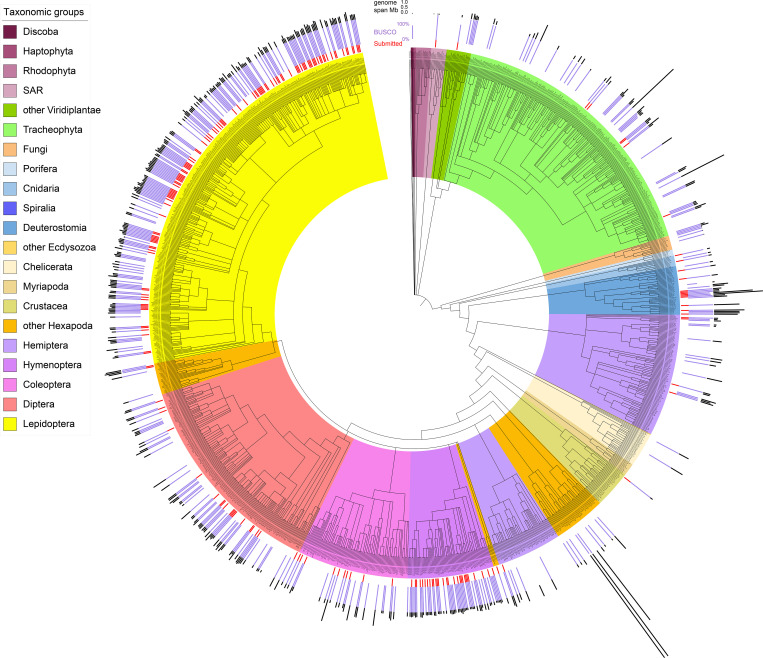
DToL: sequencing across the diversity of eukaryotes. A terabase of genomes: Eukaryotic species sequenced or undergoing sequencing in DToL to October 2021. The outer circle (black) shows estimated genome size and the middle ring (purple) the BUSCO (Eukaryota ortholog set) completeness of preliminary assemblies. The inner ring (red) shows the 200 species for which genomes have already been submitted to the ENA by DToL. The tree shows species relationships from the National Center for Biotechnology Information TaxonomyDB (https://www.ncbi.nlm.nih.gov/taxonomy) obtained using ETE (http://etetoolkit.org/). The figure was generated using IToL (https://itol.embl.de/) and postprocessed in Adobe Illustrator. The tree is available at https://itol.embl.de/shared/mblaxter2.

In the first 3 y of our project (2019 to 2022) we aim to collect specimens for 8,000 species, including—wherever possible—one for each of the ∼4,200 taxonomic families. We intend to sequence and release at least 2,000 high-quality genomes by the end of 2022. By September 2021, the DToL partnership had collected specimens for over 3,000 species, and 1,864 of these—representing 663 families—had been shipped to the Sanger Institute. Sequence data had been generated from 1,223 species, representing 348 taxonomic families, and preliminary assemblies spanning over a terabase produced from 950 species from 214 families (Fig. 2). The first 200 chromosomally complete assemblies have been submitted to the ENA (https://portal.darwintreeoflife.org/data?tracking-status=Assemblies%20-%20Submitted), and the first 23 have been annotated and are presented in Ensembl (http://projects.ensembl.org/darwin-tree-of-life/). These efforts set the stage for the second phase of our work: to sequence a representative for each family and then for each of the ∼20,000 genera in these islands. DToL genome data will be foundational for biological research for the century to come.

## Supplementary Material

Supplementary File

## Data Availability

The tree illustrating progress in sequencing of species in DToL is available from the Interactive Tree of Life (https://itol.embl.de/shared/mblaxter2). There are no other data underlying this work.
