# Supplementary material for: Sequence locally, think globally: The Darwin Tree of Life Project
Source: Proc Natl Acad Sci U S A. 2022 Jan 18;119(4):e2115642118. doi: 10.1073/pnas.2115642118 (PMC8797607; doi:10.1073/pnas.2115642118)
Supplement: Supplementary File [file pnas.2115642118.sapp01.pdf]

Darwin Tree of Life Consortium author list

Mark Blaxter[1], Nova Mieszkowska[2,3], Federica Di Palma[4], Peter Holland[5], Richard Durbin[1,6], Thomas Richards[5], Matthew Berriman[1], Paul Kersey[7], Peter Hollingsworth[8], Willie Wilson[2,9], Alex Twyford[8,10], Ester Gaya[7], Mara Lawniczak[1], Owen Lewis[5], Gavin Broad[11], Kevin Howe[12], Michelle Hart[8], Paul Flicek[12], Ian Barnes[11]

[1]Wellcome Sanger Institute, Wellcome Genome Campus, Hinxton, Cambridgeshire CB10 1SA, UK

[2]Marine Biological Association of the United Kingdom, Citadel Hill, Plymouth PL1 2PB, UK

[3]University of Liverpool, Liverpool L69 3BX, UK

[4]University of East Anglia, Norwich Research Park, Norwich NR4 7TJ, UK

[5]Department of Zoology, University of Oxford, Mansfield Road, Oxford OX1 3SZ, UK

[6]Department of Genetics, University of Cambridge, Cambridge CB2 3EH, UK

[7]Royal Botanic Gardens, Kew, Richmond, London TW9 3AE, UK

[8]Royal Botanic Garden Edinburgh, Edinburgh EH3 5LR

[9]University of Plymouth, Drake Circus, Plymouth PL4 8AA, UK

[10]Institute of Evolutionary Biology, School of Biological Sciences, University of Edinburgh, Edinburgh EH8 9YL

[11]Natural History Museum, Cromwell Road, London SW7 5BD, UK

[12]EMBL-EBI, Wellcome Genome Campus, Hinxton, Cambridgeshire CB10 1SD, UK
